# Diarrhea in the Returning Traveler: A Simulation Case for Medical Students to Learn About Global Health

**DOI:** 10.15766/mep_2374-8265.10935

**Published:** 2020-08-12

**Authors:** Zoe Lawrence, Demian Szyld, Renee Williams

**Affiliations:** 1 Resident, Department of Medicine, NYU Langone Health; 2 Lecturer, Emergency Medicine, Harvard Medical School; Attending Physician, Emergency Medicine, Brigham and Women's Hospital; Senior Director, Institute for Medical Simulation; 3 Assistant Professor, Department of Medicine, NYU Langone Health; Program Director, Gastroenterology and Hepatology Fellowship, NYU Langone Health

**Keywords:** Simulation, Gastroenterology, Clerkship, Global Health

## Abstract

**Introduction:**

As global travel becomes more prevalent, medical students may be asked to care for patients with unforeseen exposures. We developed a simulation where clerkship medical students interviewed and examined a patient with recent travel who presented with bloody diarrhea and abdominal pain and was diagnosed with amebic colitis. The students had the opportunity to develop a differential diagnosis and discuss the workup of the patient.

**Methods:**

We divided students into two groups. Each group took a turn participating in the simulation while the other group observed. Students were expected to interview and examine the patient as well as treat any urgent findings and develop a differential diagnosis. After each simulation, we reconvened with both groups for a faculty-led debriefing session to discuss the learning objectives, including approaches to caring for a patient with diarrhea and the differential diagnosis and workup of bloody diarrhea.

**Results:**

To date, five different groups of six to 12 students have completed this simulation. The module has been well received, and 100% of survey respondents have agreed that after completing the activity, they had a better understanding of how to approach a recent traveler with diarrhea and abdominal pain.

**Discussion:**

While most medical students will not travel abroad for traditional global health experiences, many will encounter patients with recent travel or immigration and must therefore be prepared to treat diseases typically categorized as global health. We developed this simulation and successfully incorporated workup of a returning traveler into the medical school curriculum for clerkship students.

## Educational Objectives

By the end of this activity, learners will be able to:

1. Elicit a relevant history in a patient presenting with bloody diarrhea and abdominal pain (including travel history, ischemic risk factors, chronicity, character of the blood, etc.).
2. Identify signs of dehydration and treat accordingly.
3. Identify signs of hypoglycemia and treat accordingly.
4. Identify electrolyte abnormalities associated with significant diarrhea.
5. Discuss the differential diagnosis in a recent traveler with bloody diarrhea and abdominal pain.
6. Discuss the workup of traveler's diarrhea.
7. Develop an understanding of entamoeba histolytica.

## Introduction

In patients with recent travel, the chief complaint of bloody diarrhea has a broader differential diagnosis than in a patient with no travel exposures. Medical students who work with a medically underserved or diverse patient population will likely encounter patients with recent travel or immigration. And although medical students are likely to learn about and treat patients with diarrhea from a multitude of etiologies, the frequency with which amebic colitis is encountered in the developed world is much less than other etiologies of bloody diarrhea.^1^ However, in interviewing, examining, and treating patients with exposures to the developing world, trainees should be aware of disease processes that are typically taught under the framework of global health. In this simulation, clerkship medical students had the opportunity to interview, examine, and stabilize a patient with amebic colitis. In participating in this exercise, learners needed to identify the sequelae and risks associated with diarrhea, including dehydration, as well as to evaluate and develop a differential diagnosis for bloody diarrhea with the patient's global and local exposures in mind.

Educational case studies involving recent travelers have been well received by medical students as they address this curricular gap surrounding global health at home.^2^ Simulation has been shown to be useful in global health education, particularly in the fields of ethics and dermatology, as well as in gastroenterology education.^3–5^ Prior modules have made use of standardized patient simulations and team-based learning activities as tools for teaching the approach to diarrhea.^6,7^ However, there is limited data on the use of manikin-based simulations as an educational and assessment tool for global health curricula, despite evidence that both manikin-based and standardized patient–based learning methods are effective and well received.^8^ There is, therefore, a need for simulation-based learning focused on global health within the standard medical school curriculum, particularly for medical students at hospitals that serve diverse, multiethnic populations. This module addressed this gap by providing students with the opportunity to triage, treat, and reflect on the case of a patient with recent travel to the developing world. The local low prevalence of patients such as this one added a level of difficulty to the module. Additionally, the module created a safe space to learn about and discuss a range of teaching points regarding bloody diarrhea.

## Methods

### Development

This simulation was implemented as part of a global health selective for students in the clerkship year of medical school. This selective was an optional course for students with an interest in global health, particularly relating to infectious diseases, noncommunicable diseases, and mental health. Within the selective, there was a focus on providing better health care to underserved populations around the world, and students had the opportunity to participate in workshops, simulations, and case discussions, as well as to gain experience in both inpatient and outpatient infectious diseases clinics.

### Equipment/Environment

• Simulation mannequin.
• Bedpan with bloody diarrhea (chunky peanut butter and jam; applesauce can be used instead of peanut butter in case of allergies).
• Sphygmomanometer.
• NG tube.
• IV catheters: 20G, 22G.
• Normal saline.
• Lactated ringers.
• Defibrillator monitors.
• ECG.
• Glucometer.
• Supplies for labs and cultures.
• D50, D10, IV potassium, magnesium, calcium, and bicarbonate solutions.
• Antidiarrheal.
• Antiemetic.
• IV antibiotics.
• Foley catheters.

### Personnel

The following personnel were needed to implement this case:

• A faculty member was the voice of the simulation mannequin and led the debrief.
• A standardized nurse (SN).
• Simulation staff member.

### Implementation

The materials for the simulation included the simulation case template (Appendix A), the student guide (Appendix B), the faculty guide (Appendix C), and the evaluation that the students were asked to fill out anonymously at the conclusion of the activity (Appendix D). Additionally, SNs were provided with the patient's laboratory results (Appendix E) and an SN guide (Appendix F).

Prior to the day of the activity, students were provided with the student guide to learn about the patient's history and the setup of the simulation day. They were encouraged to use this information to prepare by creating a preliminary differential diagnosis.

The entire curriculum took 2–3 hours. Upon arrival, the students were divided into two groups. Group one participated in the simulation while group two observed. The two groups then joined together for a debriefing session led by the faculty member. After the debriefing session, learners exchanged roles, and group two participated in the same simulation while group one observed. After the second portion, both groups again joined together for a second faculty-led debriefing session (Table 1).

**Table 1.**
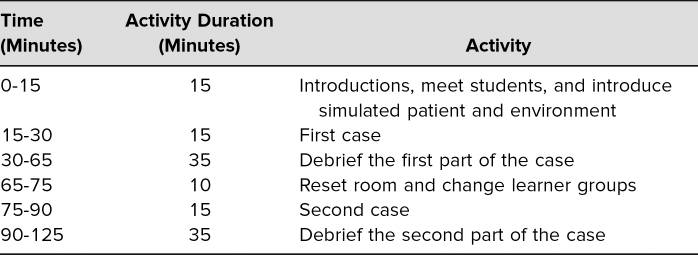
Recommended Schedule

In the simulation center, the faculty member who led the simulation operated a mechanically simulated patient. An SN was given a full list of the materials available to the students in the simulation room, and if the students requested any of those materials, the SN provided them.

### Debriefing Sessions

Each structured debriefing session was scheduled for 30–45 minutes and was composed of three phases: reactions, analysis and understanding, and summary. The sessions began with an opportunity for students to share their experiences and reactions to the exercise. In this first phase, there was time to discuss the case, the students’ feelings on how the case went, and their feedback for each other with the group who observed providing feedback to the group who participated in the simulation. This phase was followed by a faculty-led discussion centered on the learning objectives and a review of the critical actions that should have been taken. The faculty leader was provided with guidelines on what material should be covered and was able to use this opportunity to teach and to engage and guide students in self-directed learning. Learning objectives focused on both clinical care and how to best treat this patient in the moment, as well as on the differential diagnosis and workup of the patient. Finally, in the summary phase, students were asked to summarize their take-home points and process how they would approach this case differently in the future (Appendix C).

### Assessment

At the conclusion of the activity, students were asked to fill out an anonymous survey to assess their experience (Appendix D). The survey included multiple-choice questions that sought to assess how well the students felt the activity met its objectives, as well as a space to write in additional comments and feedback.

## Results

To date, five different groups of six to 12 students have completed this simulation. The number of participants has varied depending on the enrollment in the global health selective, and the estimated number of participants is 35. The entire simulation took about 3 hours to complete. Between sessions, several changes were made to the simulation in order to accommodate student and faculty feedback. On the most recent two occasions, participants filled out the aforementioned survey.

A total of 14 students completed the survey—only one student did not. All 14 students experienced the same initial simulation. Of the survey respondents, 13 (92.9%) either agreed or strongly agreed that the learning objectives were met and that the scenario was clinically relevant. Fourteen (100%) of the survey respondents either agreed or strongly agreed that at the completion of the activity, they had a better understanding of how to approach a recent traveler with diarrhea and abdominal pain and that their understanding of the diagnosis and treatment of amebic colitis was improved. When asked if they found the module worthwhile, 12 (85.7%) either agreed or strongly agreed, while two (14.3%) were neutral; no one disagreed (Table 2).

**Table 2.**
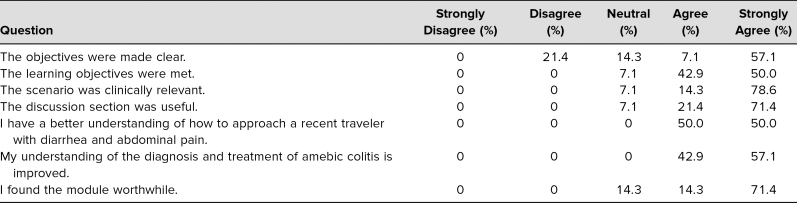
Postmodule Participant Survey (*N* = 14)

Only seven (50.0%) of the survey respondents included additional comments in the space provided on the survey. Those students wrote positive comments such as “good at creating safe learning environment,” “well organized, good teaching points,” and “very helpful case,” further solidifying that this module was well received by the participants.

## Discussion

The clerkship medical school curriculum at our institution did not include consistent exposure to patients with global exposures, despite the fact that our hospitals serve a large international population. To address this gap in the curriculum, we developed a simulation for medical students in order to provide the opportunity to evaluate and treat a patient whose illness was related to his global exposures. This simulation successfully provided clerkship medical students with the opportunity to evaluate a patient with diarrhea and recent international travel and to synthesize how the patient's travel history impacted the differential diagnosis. The students provided positive feedback for this module and uniformly felt that, by the end of the activity, they had an improved understanding of how to approach a recent traveler with diarrhea and abdominal pain.

Although some of the students did not reach the final diagnosis, the debriefing session allowed for time to learn from their mistakes and develop the confidence to manage similar patients appropriately going forward in their training and careers. Initially, our simulation had a second case in which a low-resource setting was simulated. In our experience, this second case caused confusion regarding the learning objectives and did not significantly augment the learners’ experience or understanding. The case was modified accordingly, and we found that allowing the second group of students to experience the same simulation with the benefits of having already seen their colleagues care for the patient and having participated in the first debrief session facilitated an improved comprehension. This redo of the scenario allowed for synthesis and application of educational material.

We were able to adhere to the time frame and complete this simulation in 2 hours; however, the length of the debriefing sessions will vary depending on the faculty member leading the session and the students participating. At our center, a mechanically simulated patient was used, but this simulation could be adopted to use a standardized patient instead. We chose to incorporate an SN instead of standardized patient as this role was easier to act and we did not have funding to hire a professional actor; gastroenterology fellows played the SN. Regardless, this module is relatively resource intensive, as most simulations are, which may limit its utility in some circumstances.

During clerkship year, the curriculum is focused on building the students’ performance in a clinical setting. Although lectures allow for coverage of a large amount of material delivered to a large number of learners, this type of instructional format does not test the application of skills and knowledge. Simulation-based education is an effective complement to clinical training.^9^ Furthermore, debriefing activities such as the one included in this module promote reflection and development of metacognitive skills.^10^ Because simulation and debriefing allow for development of deliberate practice styles in a safe and controlled environment, they constitute an excellent curricular tool to advance the skill set of the clerkship medical student.

Medical school global health curricula at this education level have previously been limited to international experiences that may not be observed by faculty from the students’ medical school. This module demonstrates the feasibility and applicability of a locally based global health curriculum where learners can actively use their global health clinical skills within the home educational environment and therefore receive structured and relevant feedback in real time.

Limitations of this simulation include the small scope of implementation. To date, the simulation has been implemented at only one medical school, and the results may not be generalizable to other medical schools with different patient populations and different student demographics. Furthermore, different institutions may have patients and providers who travel to areas of the world where diarrheal illnesses are not endemic but other disease processes are seen more commonly. In this situation, the outline of the simulation could be applied towards a different disease process.

The simulation is also limited by the evaluation scheme, which is based on students’ perceptions only and does not necessarily assess meaningful outcomes such as the students’ knowledge and performance. The objectives, which are skills based, are not directly assessed. Future iterations should consider revising the evaluation to incorporate faculty evaluation of the students’ performance as related to the objectives. Additionally, the evaluation of the students’ perceptions was not provided in the initial sessions of this simulation and was only added to the activity during the later sessions. As such, the opinions of a majority of the participants were not recorded, and thus, conclusions are quite limited.

All simulated clinical experiences have inherent limitations, as they do not represent true patient encounters. Standardized patients, or nurses in this case, are prepared for their role and the scenario, which may influence their responses. Furthermore, mechanically simulated patients, although quite technologically advanced, are not a true-to-life substitute and cannot portray physical exam findings with complete accuracy. While in the simulation center, learners are aware of being watched and are thus likely to feel self-conscious. This feeling may result in the learners performing differently than they would in a normal clinical encounter, and it may affect their learning and their experience as well as their perception of the activity.

Despite these limitations, this simulation provided an opportunity to incorporate global health into the third-year medical school curriculum. While most medical students will not travel abroad to have traditional global health experiences, many, if not all, will encounter patients with recent travel or immigration and must therefore be prepared to treat diseases typically categorized as global health. Using modules such as this one allows medical students to have global health experience without leaving their home country.

## Appendices

Simulation Case Template.docxStudent Guide.docxFaculty Guide.docxEvaluation.docxLaboratory Values.docxStandardized Nurse Guide.docx All appendices are peer reviewed as integral parts of the Original Publication.
