# Supplementary material for: Diarrhea in the Returning Traveler: A Simulation Case for Medical Students to Learn About Global Health
Source: MedEdPORTAL. 2020 Aug 12;16:10935. doi: 10.15766/mep_2374-8265.10935 (PMC7431184; doi:10.15766/mep_2374-8265.10935)
Supplement: Supplementary file 1 — Simulation Case Template.docxStudent Guide.docxFaculty Guide.docxEvaluation.docxLaboratory Values.docxStandardized Nurse Guide.docx [file mep_2374-8265.10935-s001.zip › A. Simulation Case Template.docx]

| **Appendix A: MedEdPORTAL Simulation Case Template**  **SIMULATION CASE TITLE:** Diarrhea in the Returning Traveler: A Simulation Case for Medical Students to Learn about Global Health  **AUTHORS: Zoe Lawrence, Demian Szyld, Renee Williams**  **LEARNER AUDIENCE: clerkship medical students** | |
| --- | --- |
| **PATIENT NAME: Mr. Smith**  **PATIENT AGE: 27 years**  **CHIEF COMPLAINT: abdominal pain**  **PHYSICAL SETTING: emergency room** | |
|  | |
| **Brief narrative description of case** | Mr. Smith is a 27-year-old male who presents with complaints of abdominal pain and diarrhea. Half the students will participate in the simulation while the other half observe, then all students will join together for a debriefing session. In the second portion of the case, the groups change roles so that the observers have a chance to participate in the simulation before again joining up for a second debriefing session with all learners. Overall learn goals are to develop a team-based approach to assess the patient, to evaluate the patient, to treat any urgent findings, and to develop a differential diagnosis that will be communicated with the attending and the patient. |
| **Primary Learning Objectives** | 1. Elicit a relevant history in a patient presenting with bloody diarrhea and abdominal pain (including travel history, ischemic risk factors, chronicity, character of the blood, etc). 2. Identify signs of dehydration and treat accordingly 3. Identify signs of hypoglycemia and treat accordingly 4. Identify electrolyte abnormalities associated with significant diarrhea 5. Discuss the differential diagnosis in a recent traveler with bloody diarrhea and abdominal pain 6. Discuss the workup of traveler’s diarrhea 7. Develop an understanding of entamoeba histolytica |
| **Critical Actions** | 1. Insert short, large bore IV’s 2. Check capillary glucose and treat hypoglycemia with D50 3. Send appropriate labs including basic metabolic panel and a complete blood count. Results will be available quickly 4. Note hypokalemia, anemia, and leukocytosis 5. Promptly initiate IV fluid resuscitation based on tachycardia and hypotension (orthostatics) 6. Identify at least three potential diagnoses |
| **Learner Preparation or Prework** | The learners should be presented with the following information prior to the initiation of the case.  Situation: Mr. Smith is a 27-year-old man who presents with complaints of abdominal pain and diarrhea.  Background: He has a history of alcohol use disorder but has not used in over 2 years. He has no known allergies and he is full code. On presentation, his vital signs are Pulse 110, BP 90/60, T 101F, SpO2 99% on room air. The patient was evaluated in urgent care and determined to need further evaluation in the emergency room.  Instructions:  - Join your team in the emergency room  - As a team, review the briefing regarding the patient  - Interview and evaluate the patient  - Provide basic treatment for any urgent findings (including ordering labs, responding to lab abnormalities, and ordering basic medications)  - Develop a differential diagnosis  - Communicate the plan with the patient and the attending |

| Initial Presentation | | | |
| --- | --- | --- | --- |
| **Initial vital signs** | Pulse 110, BP 90/60, T 101F, SpO2 99% on room air | | |
| **Overall Setting and Appearance** | The room set is set up to resemble an emergency room bay. Upon entering the room, learners will see a standardized nurse (SN) and a simulation mannequin. Next to the bedside is a bedpan filled with bloody diarrhea, which is made from chunky peanut butter and jam mixed together. (If there is a concern for peanut allergy, the bloody stool can also be made from apple sauce mixed with jam.) | | |
| **Confederates (e.g., standardized participants) and their roles in the room at case start** | The SN played by any provider or actor is present in the room at the beginning with the simulation mannequin.  When the students enter the room, the SN will provide the following introduction:  “You are rotating through the Emergency Room. The Attending physician has been called away for a trauma and you are asked to see Mr. Smith who presents with complaints of diarrhea and abdominal pain. I will be a nurse at the bedside. You can ask me for supplemental information such as labs or about the physical exam if aspects are not clear from examining the patient.  “Your task will be to obtain a detailed history on the patient and perform a physical exam based on the presenting complaint. You are also expected to make management decisions depending on the scenario. You will be expected to present your information and your interpretation of the case to the Attending physician at the conclusion of the visit.”  The SN will then pause for questions before letting the students know that the simulation is beginning by saying  “Hi I am the nurse taking care of the patient; the Attending physician is unavailable and would like to evaluate Mr. Smith. He is a 27 year old male presenting with diarrhea and abdominal pain.“ | | |
| **HPI** | 27-year-old male with a past medical history of alcohol use disorder, now abstinent, who presents for evaluation of diarrhea for 2 months. He traveled to India for two months on a study elective, while in India he drank local water and ate street food. His symptoms started about two months ago while still in India. He started experiencing vague abdominal cramping that would worsen with eating. When he returned to the USA he started having multiple small volume non-bloody diarrhea preceded by his abdominal cramping. After one week he started to pass red blood with his bowel movements. He has an average of 5 bowel movements a day.  He had been to the ER twice for the same symptoms and has had labs and stool studies drawn. He also has some nausea along with multiple episodes of emesis.  He has lost a significant amount of weight in the past few months and doesn’t really have an appetite.  If the students ask for orthostatic vitals, the nurse can share that the blood pressure was 90/60 lying down and 55/20 sitting up.  All other information can be volunteered by the patient in response to appropriate questioning by the students. | | |
| **Past Medical/Surgical History** | **Medications** | **Allergies** | **Family History** |
| Alcohol use disorder, sober for 2 years | None | No known drug allergies | Negative |
| **Physical Examination** | | | |
| **General** | Appears well nourished and well developed | | |
| **HEENT** | Dry mucous membranes | | |
| **Neck** | No lymphadenopathy, no jugular venous distention | | |
| **Lungs** | Clear to auscultation bilaterally | | |
| **Cardiovascular** | Normal s1/s2, no murmurs/rubs/gallops | | |
| **Abdomen** | Soft but distended, diffusely tender to palpation. No rebound or guarding. Hyperactive bowel sounds. | | |
| **Neurological** | hyporeflexia | | |
| **Skin** | Tenting | | |
| **Rectal** | Brown stool with red blood mixed in | | |
| **Psychiatric** | Alert and oriented, answers all questions appropriately | | |

| Instructor Notes - Changes and CASE Branch Points | | |
| --- | --- | --- |
| **Intervention / Time point** | **Change in Case** | **Additional Information** |
| 3 minutes into the case | BP begins decreasing if no IV fluids have been given for hypotension | RN alerts the provider: “Doctor, the blood pressure is 80/45” |
| 4 minutes into the case | Patient complains of nausea and diaphoresis if the capillary glucose has not been checked | Patient states: “I feel really nauseated and sweaty and I haven’t eaten in days” |
| Participant administers fluids | Blood pressure increases to 100/70 and HR decreases to 95 | Patient states “I think the fluid is helping me” |
| Participant requests finger stick blood glucose. | Glucose level is 40. | Glucose level will improve if patient is given IV D50. |
| Participant requests a basic metabolic panel | Potassium level is 3.3 | Patient reports some improvement in symptoms if KCl is administered |
| 5 minutes into the case | Patient complains of nausea | Nausea will resolve if anti-emetics are administered |

**Ideal Scenario Flow**

The learners enter the room to find a patient who is uncomfortable appearing and is complaining of nausea. They begin to gather the history and discover that he has been experiencing bloody diarrhea, abdominal pain, and nausea/vomiting for the past 5 weeks since he returned from a trip to India. The learners will check his vital signs, which are displayed on a computer and react by asking the SN to place an IV and administer fluids. The learners will begin to examine the patient and then ask the SN to draw basic labs including a basic metabolic panel. The patient will complain of nausea and diaphoresis at which point the learners will administer anti-emetics and ask for a capillary glucose. The capillary glucose level is 40 and the learners will administer D50 to good effect. The labs return and the potassium level is 3.3. The learners acknowledge that the potassium level is low. Once the fluids have finished running, the heart rate will decrease and the blood pressure will increase. At the end of the case, the faculty member will enter the room and introduce him/herself and ask one of the students to present the case and their differential diagnosis.

Following the first debriefing session, the learners will change roles and the group that participated in the scenario will step out and observe while the group that had previously observed has the opportunity to participate in the simulation. All other aspects of the case remain the same.

**Anticipated Management Mistakes**

1. *Failure to recognize the need for IV fluids: Some of our learners did not immediately recognize that the patient was significant dehydrated and required IV fluid. We found it helpful to allow the blood pressure to decrease a few minutes into the scenario in order to prompt the need for fluid resuscitation.*
2. *Lack of familiarity with amebic colitis: Many of our learners were unfamiliar with amebic colitis. We created specific debriefing materials to address this knowledge gap.*
