# Supplementary material for: Diarrhea in the Returning Traveler: A Simulation Case for Medical Students to Learn About Global Health
Source: MedEdPORTAL. 2020 Aug 12;16:10935. doi: 10.15766/mep_2374-8265.10935 (PMC7431184; doi:10.15766/mep_2374-8265.10935)
Supplement: Supplementary file 1 — Simulation Case Template.docxStudent Guide.docxFaculty Guide.docxEvaluation.docxLaboratory Values.docxStandardized Nurse Guide.docx [file mep_2374-8265.10935-s001.zip › B. Student Guide.docx]

Diarrhea in the Returning Traveler: A Simulation Case for Medical Students to Learn about Global Health

**Mr. Smith**

| **Situation:** | Mr. Smith is a 27-year-old male who presents with complaints of abdominal pain and diarrhea. |
| --- | --- |
| **Background:** | He has a history of alcohol use disorder but has not used in over 2 years. He has no known allergies. Code status: Full.  On presentation his vital signs are:  Pulse 110; BP 90/60; T 101; RR 20; 02 Sat 99%RA |
| **Assessment:** | The patient was evaluated in urgent care and determined to need further evaluation in the emergency room. |
| **Instructions:** | - Join your team in the emergency room  - As a team, review the briefing regarding the patient  - Interview and evaluate the patient  - Provide basic treatment for any urgent findings (including ordering labs, responding to lab abnormalities, and ordering basic medications)  - Develop a differential diagnosis  - Communicate the plan with the patient and the attending |

*please advise your faculty leader prior to the activity if you are allergic to peanuts
