# Supplementary material for: Diarrhea in the Returning Traveler: A Simulation Case for Medical Students to Learn About Global Health
Source: MedEdPORTAL. 2020 Aug 12;16:10935. doi: 10.15766/mep_2374-8265.10935 (PMC7431184; doi:10.15766/mep_2374-8265.10935)
Supplement: Supplementary file 1 — Simulation Case Template.docxStudent Guide.docxFaculty Guide.docxEvaluation.docxLaboratory Values.docxStandardized Nurse Guide.docx [file mep_2374-8265.10935-s001.zip › C. Faculty Guide.docx]

Diarrhea in the Returning Traveler: A Simulation Case for Medical Students to Learn about Global Health

Welcome to the simulation center. This simulation is a part of the Global Health Selective. The students are currently in their clerkship year of medical school. The focus of this exercise will be evaluating, managing, and diagnosing a patient with bloody diarrhea and a recent history of international travel.

• There will be one group of 6 to 12 students

• All the students will actively participate in both debriefings. This scenario includes two parts, as such 3-6 students will do the first portion of the case and the next 3-6 will perform the second half of the case.

• The group not participating in the case will observe and take notes to give feedback during debriefing sessions.

• You will have 3 hours for the session

Objectives (Part 1):

1. Elicit a relevant history in a patient presenting with bloody diarrhea and abdominal pain (including travel history, ischemic risk factors, chronicity, character of the blood, etc).
2. Identify signs of dehydration and treat accordingly
3. Identify signs of hypoglycemia and treat accordingly
4. Identify electrolyte abnormalities associated with significant diarrhea

Objectives (Part 2)

1. Discuss the differential diagnosis in a recent traveler with bloody diarrhea and abdominal pain
2. Discuss the workup of traveler’s diarrhea
3. Develop an understanding of entamoeba histolytica

**Recommended Schedule**

| **Time** | **Minutes** | **Activity** |
| --- | --- | --- |
| 0-15 | 15 | Introductions, meet students, and introduce simulated patient and environment. |
| 15-30 | 15 | First case |
| 30-65 | 35 | Debrief first part of the case |
| 65-75 | 10 | Reset room and change learner groups |
| 75-90 | 15 | Second case |
| 90-125 | 35 | Debrief the second part of the case |

**Introduction to students**

• “Today you will care for a simulated patient. These mechanical simulators blink, breathe, talk, and have pulses, vital signs and realistic physiology.”

• Please show the learners the following features of the simulated patients

1. The patient can provide a history and can be examined for physical findings; I will be the voice of this patient.
2. There will be someone playing the role of a nurse in the room. Whenever the technology falls short, the standardized nurse (SN) will fill in with information. For example most of the neurological and skin part of the exam.
3. The patient will blink when awake and close his eyes when sleeping/unconscious.
4. The patient has a palpable carotid, femoral and pedal pulses
5. The patient has audible heart sounds, lung sounds and abdominal sounds
6. This patient can tolerate a nasogastric (NG) tube, intubation with an endotracheal tube, IV access, needle decompression, defibrillation via metal electrodes. The patient can be monitored with a pulsoximter and 3 lead ECG. You can measure a systolic and diastolic blood pressure.
7. On the screen above we can show you radiology images

• Please remember that simulation is not 100% real. This allows us to reliably represent rare scenarios… and on the other hand has its limitations – you may not perform exactly as you would in “the real world.” Nonetheless it would help us talk about interesting and important topics.

• To send labs, you just need to tell the SN what you are sending

•To administer medications, you must use an air filled syringe that is labeled. You can give IV, IM in this fashion. For oral medications and IV drips, simply state what you are giving. Don’t forget to note the dose and route of administration.

**The Case:**

Present this case by introducing yourself as the attending in the Emergency Room at a city hospital. The students are working as a team independently. You are unavailable however they will have a nurse available to answer questions throughout the case. They should see the patient, treat any emergent findings, and then develop a differential diagnosis to discuss with you.

Time to get started -- allow the first group of students to enter the room.

**Debriefing/Discussion**

•**Intro**

– Inform the learners how you plan to conduct this debriefing

**Phase 1: Reactions**

**Phase 2: Analysis and understanding**

**Phase 3: Summary**

**Reactions phase:**

1) Ask the students how did that feel?

2) Ask any student to volunteer to share their impression or summary of what the main points of the case were about.

3) Complement their summary with anything they might have missed.

**Analysis and understanding phase:**

Lead a discussion on the following learning objectives:

1. Elicit a relevant history in a patient presenting with bloody diarrhea and abdominal pain
   1. Time course: is this patient compensated and has been living with weeks or months of bloody diarrhea or is this a sudden onset, hemodynamically significant change from baseline? This distinction will help you triage the patient between the clinic and the hospital
   2. Is the blood mixed in the stool, only on the outside of the stool, or separate from the stool and turning the toilet bowl water red? Each of these findings is associated with different types of pathology
   3. Exposures such as travel, animals, and new foods can help guide the differential diagnosis and make certain diagnoses more or less likely. This patient’s travel history makes infectious colitis more likely. It is important to be wary of implicit biases – always ask about exposures and make no assumptions.
2. Identify signs of dehydration and treat accordingly
   1. The patient is orthostatic, hypotensive and tachycardic
   2. He also has an elevated Creatinine
   3. He needs resuscitation with an isotonic solution such as lactated ringers or normal saline
3. Identify signs of hypoglycemia and treat accordingly
   1. Patient is diaphoretic and complains of nausea
   2. Blood sugar is low
   3. Treatment requires giving the patient glucose, and since he is nauseated, it is preferable to give glucose IV rather than PO
4. Identify electrolyte abnormalities associated with significant diarrhea
   1. Hypokalemia secondary to emesis and fluid losses of his diarrhea
   2. Elevated creatinine secondary to dehydration
   3. Hypoglycemia secondary to poor intake

Critical Actions checklist:

Give feedback on whether the students performed the following critical actions

1. Insert short, large bore IV’s
2. Check capillary glucose and treat hypoglycemia with D50
3. Send appropriate labs including basic metabolic panel and a complete blood count
4. Note hypokalemia, anemia, and leukocytosis
5. Promptly initiate IV fluid resuscitation based on tachycardia and hypotension (orthostatics)
6. Develop a differential diagnosis of at least three diagnoses

**Summary Phase**

Ask the students to summarize their take home points from this case. Of the thought processes and actions, which would you repeat in the future? What would you do or think differently next time?

**Second portion of the case**

The group that observed the first portion will now participate in the same simulation while the first group observes.

All aspects of the case remain the same.

**Debriefing/Discussion points (Part 2):**

**Phase 1: Reactions**

**Phase 2: Analysis and understanding**

**Phase 3: Summary**

**Reactions phase:**

1) Ask the students how did that feel?

2) Ask any student to volunteer to share their impression or summary of what the main points of the case were about.

3) Complement their summary with anything they might have missed.

**Analysis and understanding phase:**

Lead a discussion on the following learning objectives:

1. Discuss the differential diagnosis in a recent traveler with bloody diarrhea and abdominal pain

- 1. Infectious etiologies must always be considered in bloody diarrhea, but particularly in someone with international travel to underdeveloped areas, infections should be the top of the differential
     1. Infectious diarrhea with grossly bloody stools can be caused by shigella, salmonella, campylobacter, EHEC, and amebic colitis^1^
  2. Ischemia (mesenteric ischemia and ischemic colitis) are considerations but are less likely in this otherwise healthy young man without vascular risk factors
  3. Inflammatory bowel disease (Crohn’s and Ulcerative colitis) initial presentation or flare could cause this patient’s abdominal pain and bloody stool
  4. In patients with substance use disorders, particularly alcohol use disorder, consider peptic ulcer disease, Mallory Weiss tears, or variceal bleeding. Variceal bleeding and PUD are more likely to present with melena than bright red blood per rectum, and Mallory Weiss tears are more likely to present with small volume hematemesis and so are less likely in our patient
  5. Hemorrhoids are a common cause of lower GI bleeding and must be considered in this patient, however abdominal pain and diarrhea are not typically seen
  6. Diverticular bleeding is often painless but can present with abdominal pain if associated with diverticulitis. This patient is young to have diverticular bleeding making this less likely.

1. Discuss the workup of traveler’s diarrhea
   1. The sensitivity of stool tests for traveler's diarrhea
      1. Stool culture tests for some, but not all causes of bacterial diarrhea, as such it is important for the clinician to know which pathogens are and are not detected by their laboratories culture mediums. Fever, > 5 days of diarrhea, CRP >50mg/L, and stool WBC have been shown to be independently associated with positive stool culture, and so culture may be most useful in patients with these characteristics.^2,3^
      2. Some common causes of traveler’s diarrhea are due to parasites, not bacteria. Testing stool for ova and parasites is the appropriate tool for diagnosing these illnesses. Detection of ova and parasites relies mostly on laboratory personnel using bright-field microscopy. As with culture, clinicians must be aware of which parasites are routinely tested for – additional tests must often be requested for microsporidia for example.^4^
   2. Specific indications for colonoscopy in a patient with enteroinvasive disease
      1. Colonoscopy is not usually indicated for patients with acute diarrhea. However, if stool studies are inconclusive and the patient continues to have diarrhea, or if diarrhea worsens despite empiric treatment, colonoscopy is indicated. Colonoscopy and biopsy can differentiate between inflammatory bowel disease, ischemic colitis, malignancy, and infectious diarrhea. If this patient were to have a colonoscopy, you would see small, scattered, punched out ulcers.^5^
2. Develop an understanding of entamoeba histolytica
   1. Second leading cause of death from parasitic disease worldwide.^6^
   2. Humans ingest *E. Histolytica* cysts through fecally contaminated water or food. Cysts pass into the small intestine where excystation occurs in the terminal ileum resulting in motile trophozoites. The trophozoites aggregate in the mucin layer of the colon and cecum and form new cysts, which are excreted in the feces.^1^
   3. Colitis occurs when the trophozoite invades the intestinal mucous layer and causes lysis of tissue hence the name “*histolytica.*” Disruption of this protective mucous layer causes inflammation, bleeding and diarrhea. A similar process can also cause amebic liver and brain abscesses.^1^
   4. Typically presents with onset of symptoms over several weeks. Increasingly severe diarrhea is primary complaint with abdominal pain. ^1^
   5. Treatment: Metronadizole or Tinidazole for 5-10 days plus a luminal agent such as paromycin to eliminate intraluminal cysts.^7^

Critical Actions checklist:

Give feedback on whether the students performed the following critical actions

1. Insert short, large bore IV’s
2. Check capillary glucose and treat hypoglycemia with D50
3. Send appropriate labs including basic metabolic panel and a complete blood count
4. Note hypokalemia, anemia, and leukocytosis
5. Promptly initiate IV fluid resuscitation based on tachycardia and hypotension (orthostatics)
6. Develop a differential diagnosis of at least three diagnoses (preferably not all the same as the diagnoses listed by the first group)

**Summary Phase**

Ask the students to summarize their take home points from this case. Of the thought processes and actions, which would you repeat in the future? What would you do or think differently next time?

References

1. Haque R, Huston CD, Hughes M, Houpt E, Petri WA. Amebiasis. *N. Engl. J. Med.* 2003;348(16):1565-73.

2. Lee JY, Cho SY, Hwang HSH, et al. Diagnostic yield of stool culture and predictive factors for positive culture in patients with diarrheal illness. *Medicine* 2017;96(30):e7641. doi:10.1097/MD.0000000000007641.

3. Humphries RM, Linscott AJ. Laboratory diagnosis of bacterial gastroenteritis. *Clin. Microbiol. Rev.* 2015;28(1):3-31. doi:10.1128/CMR.00073-14.

4. Garcia LS, Arrowood M, Kokoskin E, et al. Laboratory Diagnosis of Parasites from the Gastrointestinal Tract. *Clin. Microbiol. Rev.* 2018;31(1). doi:10.1128/CMR.00025-17.

5. Bhagatwala J, Singhal A, Aldrugh S, Sherid M, Sifuentes H, Sridhar S. Colonoscopy — indications and contraindications. In: Ettarh R, ed. *Screening for Colorectal Cancer with Colonoscopy*. InTech; 2015. doi:10.5772/61097.

6. Stanley Jr SL. Amoebiasis. *Lancet* 2003;361:1025-34.

7. Thielman NM, Guerrant RL. Acute Infectious Diarrhea. *N. Engl. J. Med.* 2004;350(1):38-47.
