# Supplementary material for: Diarrhea in the Returning Traveler: A Simulation Case for Medical Students to Learn About Global Health
Source: MedEdPORTAL. 2020 Aug 12;16:10935. doi: 10.15766/mep_2374-8265.10935 (PMC7431184; doi:10.15766/mep_2374-8265.10935)
Supplement: Supplementary file 1 — Simulation Case Template.docxStudent Guide.docxFaculty Guide.docxEvaluation.docxLaboratory Values.docxStandardized Nurse Guide.docx [file mep_2374-8265.10935-s001.zip › D. Evaluation.docx]

Diarrhea in the Returning Traveler: A Simulation Case for Medical Students to Learn about Global Health

Please complete the survey below. Thank you!

1. The objectives were made clear
   - Strongly disagree
   - Disagree
   - Neutral
   - Agree
   - Strongly agree
2. The learning objectives were met
   - Strongly disagree
   - Disagree
   - Neutral
   - Agree
   - Strongly agree
3. The scenario was clinically relevant
   - Strongly disagree
   - Disagree
   - Neutral
   - Agree
   - Strongly agree
4. The discussion was useful
   - Strongly disagree
   - Disagree
   - Neutral
   - Agree
   - Strongly agree
5. I have a better understanding of how to approach a recent traveler with diarrhea and abdominal pain
   - Strongly disagree
   - Disagree
   - Neutral
   - Agree
   - Strongly agree
6. My understanding of the diagnosis and treatment of amebic colitis is improved
   - Strongly disagree
   - Disagree
   - Neutral
   - Agree
   - Strongly agree
7. I found the module worthwhile
   - Strongly disagree
   - Disagree
   - Neutral
   - Agree
   - Strongly agree
8. Additional comments
