# Supplementary material for: Diarrhea in the Returning Traveler: A Simulation Case for Medical Students to Learn About Global Health
Source: MedEdPORTAL. 2020 Aug 12;16:10935. doi: 10.15766/mep_2374-8265.10935 (PMC7431184; doi:10.15766/mep_2374-8265.10935)
Supplement: Supplementary file 1 — Simulation Case Template.docxStudent Guide.docxFaculty Guide.docxEvaluation.docxLaboratory Values.docxStandardized Nurse Guide.docx [file mep_2374-8265.10935-s001.zip › E. Laboratory Values.docx]

Diarrhea in the Returning Traveler: A Simulation Case for Medical Students to Learn about Global Health

Basic Metabolic Panel

Na 135

K 3.3

Cl 108

Bun 40

Cr 1.6

Glu 40

Hepatic Function Panel

AST 20

ALT 21

Alk phos 100

T bil 0.1

D.Bil 0.4

T.P 7.0

Alb 4.0

Hemogram (CBC)

Wbc 12.6

Hgb 10.9

Hct 32.6

Plt 328

MCV 91.6
RDW 21.4

Neut % 69.6

Lymph % 17.5H

Mono 7.5

Eos % 4.9H

ABG: 7.38/32/100/20/100%

ESR normal
CRP normal

Iron 25

Normal ferritin

Normal TIBC

Stool studies:

Ova and parasites negative x 2

Fecal occult blood positive

Fecal leukocytes few

Stool culture negative

Giardia negative

Strongyloides Ab negative

C. Difficile negative

Colonoscopy:

Entire Colon: chronic active colitis with mild architectural distortion and increase in eosinophils. No evidence of granulomas, dysplasia or parasites. Would consider inflammatory bowel disease on differential.

Ileum: Normal.
