# Supplementary material for: Diarrhea in the Returning Traveler: A Simulation Case for Medical Students to Learn About Global Health
Source: MedEdPORTAL. 2020 Aug 12;16:10935. doi: 10.15766/mep_2374-8265.10935 (PMC7431184; doi:10.15766/mep_2374-8265.10935)
Supplement: Supplementary file 1 — Simulation Case Template.docxStudent Guide.docxFaculty Guide.docxEvaluation.docxLaboratory Values.docxStandardized Nurse Guide.docx [file mep_2374-8265.10935-s001.zip › F. Standardized Nurse Guide.docx]

**Diarrhea in the Returning Traveler: A Simulation Case for Medical Students to Learn about Global Health**

Thank you for agreeing to participate in the Global Health Gastroenterology simulation module. The students are currently in their clerkship year of medical school. The focus of this exercise will be their reporting, interpreting and diagnostic skills. The students are expected to manage the clinical aspects of the case.

- The students will be in groups of 6-12 students in the room at any time
- The simulation will run for 10 minutes maximum

**RECOMMENDED SCHEDULE**

| **Time** | **Minutes** | **Activity** |
| --- | --- | --- |
| 0-15 | 15 | Introductions, meet students, and introduce simulated patient and environment. |
| 15-30 | 15 | First case |
| 30-65 | 35 | Break while students debrief first part of the case |
| 65-75 | 10 | Reset room and change learner groups |
| 75-90 | 15 | Second case |
| 90-125 | 35 | Break while students debrief second part of the case |

**Introduction to Simulation: Script for standardized nurses (SN’s)**

“You are rotating through the Emergency Room. The Attending physician has been called away for a trauma and you are asked to see Mr. Smith who presents with complaints of diarrhea and abdominal pain. I will be a nurse at the bedside. You can ask me for supplemental information such as labs or about the physical exam if aspects are not clear from examining the patient.

“Your task will be to obtain a detailed history on the patient and perform a physical exam based on the presenting complaint. You are also expected to make management decisions depending on the scenario. You will be expected to present your information and your interpretation of the case to the Attending physician at the conclusion of the visit.”

*Pause for questions*

Start the simulation by saying

“Hi I am the nurse taking care of the patient; the Attending physician is unavailable and would like to evaluate Mr. Smith. He is a 27 year old male presenting with diarrhea and abdominal pain.“

Resources Available (This list does not need to be read aloud to participants)

- Sphygmomanometer
- NG tube
- IV Catheters: 20G, 22G
- Normal Saline
- Lactated Ringers
- Defibrillator Monitors
- ECG
- Glucometer
- Supplies for labs and cultures
- D50, D10, IV Potassium, Magnesium, Calcium and Bicarbonate solutions
- Anti-diarrheal
- Antiemetic
- IV Antibiotics
- Foley Catheters

**Case Introduction**

There will be a basin containing bloody diarrhea in the room-- this will be peanut butter (or apple sauce) mixed with jam.

**Allow students to gather the HPI:**

**CC**: abdominal pain

**HPI**: 27-year-old male with a past medical history of alcohol use disorder, now abstinent, who presents for evaluation of diarrhea for 2 months. He traveled to India for two months on a study elective, while in India he drank local water and ate street food. His symptoms started about two months ago while still in India. He started experiencing vague abdominal cramping that would worsen with eating. When he returned to the USA he started having multiple small volume non-bloody diarrhea preceded by his abdominal cramping. After one week he started to pass red blood with his bowel movements. He has an average of 5 bowel movements a day.

He had been to the ER twice for the same symptoms and has had labs and stool studies drawn. He also has some nausea along with multiple episodes of emesis.

He has lost a significant amount of weight in the past few months and doesn’t really have an appetite.

**Medications:** none

**Allergies:** None

**Social History:** non-smoker. Has not used alcohol in 2 years and is currently a student

**Family History**: negative

**Physical Exam:**

Don’t present the physical exam- Allow the students to do the evaluation. Add in findings as needed especially those that cannot be represented on the simulated patient (The patient is “*well-appearing”).*

If they ask for a neuro exam, state *“I noticed his reflexes were diminished”*

If they ask for a skin exam, state *“I noticed some skin tenting”*

PHYSICAL EXAM (parts of the exam not listed here are normal)

HR 110 regular RR 20 BP 90/60 laying down, (If asked) 55/20 sitting up, Temp 101F, Weight: 70 Kg

GENERAL APPEARANCE: Appears well nourished and well developed

Mental Status: Alert and oriented, answers all questions appropriately

(if asked) HEENT: dry mucous membranes

CV: normal s1/s2, no murmurs/rubs/gallops.

LUNGS: Clear to auscultation bilaterally

ABDOMEN: soft but distended, diffusely tender to palpation. No rebound or guarding. Hyperactive bowel sounds.

EXTREMITIES: no cyanosis, edema or clubbing.

(If asked) RECTAL: brown stool with red blood mixed in

(If asked) NEURO: hyporeflexia

(If asked) SKIN: tenting

**Play of the Case:**

If asked for any of the resources listed above, give them freely.

At the end of the case, the faculty member will enter the room and introduce him/her-self as the ER doctor; s/he will then ask one of the students to present their findings. At that point the nurse will state “*I need to go and see another patient*” and leave the room.

**Case 1, part 2:**

The group that observed the first portion will now participate in the same simulation while the first group observes.

All aspects of the case remain the same.
